# Rab27a Was Identified as a Prognostic Biomaker by mRNA Profiling, Correlated with Malignant Progression and Subtype Preference in Gliomas

**DOI:** 10.1371/journal.pone.0089782

**Published:** 2014-02-26

**Authors:** Hongjun Wang, Yan Zhao, Chuanbao Zhang, Mingyang Li, Chuanlu Jiang, Yongli Li

**Affiliations:** 1 Department of Neurosurgery, the Second Affiliated Hospital of Harbin Medical University, Harbin, China; 2 Department of Neurosurgery, Beijing Tiantan Hospital, Capital Medical University, Beijing, China; University of Dundee, United Kingdom

## Abstract

**Purpose:**

Rab27a belongs to the Rab small GTPase superfamily. The protein is membrane-bound and may be involved in protein transport and small GTPase-mediated signal transduction. Mutations in this gene are associated with Griscelli syndrome type 2. However, the prognostic and molecular features of gliomas with Rab27a expression are still unclear.

**Experimental Design:**

We used a whole-genome mRNA expression microarray dataset of 220 glioma samples from the Chinese Glioma Genome Atlas (CGGA) database (http://www.cgga.org.cn) as a discovery set. In this set, 220 gliomas, consisting of 97 WHO Grade II gliomas, 34 WHO Grade III gliomas, and 89 WHO Grade IV gliomas, were analyzed using the Kaplan-Meier method. To validate the protein expression of Rab27a, we assayed another 162 glioma samples by immunohistochemistry. Three additional datasets were obtained as validation sets. Gene ontology (GO) analysis and gene set variation analysis (GSVA) were used for the functional annotation of Rab27a in 89 WHO Grade IV gliomas.

**Results:**

Rab27a was significantly associated with grade progression and high mortality in all grades of glioma in the discovery set. Rab27a also showed a mesenchymal subtype, G3 subtype and isocitrate dehydrogenase 1 (IDH1) wild-type preference and association with migration. The 3 validation datasets revealed similar findings. Rab27a was more highly expressed in gliomas than in normal brain tissues, and its expression increased with glioma grade progression.

**Conclusions:**

Rab27a expression was significantly associated with grade progression and worse prognosis in all grades of gliomas, suggesting Rab27a as a novel biomarker with potentially important therapeutic implications.

## Introduction

Glioma is the most common and malignant brain tumor, but its prognosis has changed little over the past 30 years, despite comprehensive and intensive treatment. Glioblastoma (GBM) is the most malignant glioma, the median survival time of which is only approximately one year [1], [2]. The invasive capacity of GBM cells is greater than that of cells from other glioma grades (WHO Grade II and WHO Grade III gliomas), which may contribute in part to its poor prognosis. As such, there is an urgent need for research aimed at understanding glioma invasiveness.

The Rab protein is a member of the small GTPase superfamily. Rab is located in a specific subcellular organelle and plays an important role in cell secretion, endocytosis, signal transduction, and development [3]. Rab27a is the only member of the Rab family whose mutation has been documented to result in a hereditary disease, specifically, Griscelli syndrome. Recently, some reports have shown that Rab27a is associated with a series of cancers and that Rab27a expression is closely correlated with tumor progression. Rab27a was also shown to be a valuable prognostic indicator for hepatocellular carcinoma patients and is well known to be a driver of melanoma, bladder cancer, and breast cancer by promoting cell proliferation, invasion, and metastasis. [4], [5], [6]. So far, a few reports have shown that Rab27a can promote proliferation and invasion and can repress apoptosis. However, these studies were only based on functional assays in a glioma cell line [7], [8]; the comprehensive expression patterns and effect of Rab27a on the occurrence and development of glioma are still unclear.

In this study, we analyzed mRNA expression microarray data from 220 samples comprising all grades of glioma from the Chinese Glioma Genome Atlas (CGGA) database as the discovery set. We then analyzed 3 additional published datasets as validation sets. After evaluating the expression level of Rab27a in these samples, we assessed its prognostic value. The expression pattern of Rab27a was validated by immunohistochemistry in another 162 glioma samples from the Chinese Glioma Tissue Database (CGTD). We also performed function annotation of Rab27a by gene ontology (GO) analysis and gene set variation analysis (GSVA) in all 4 datasets and discovered a correlation between its expression and cell migration.

## Materials and Methods

### Datasets used in this Study

Whole-genome mRNA expression microarray data and clinical information from 220 glioma samples of all grades from the Chinese Glioma Genome Atlas (CGGA) database [1] (http://www.cgga.org.cn) were used as the discovery set. A total of 220 frozen glioma samples were obtained from newly diagnosed patients treated by the CGGA Group. Two neuropathologists independently confirmed the tumor histologies from all patients; confirmation was based on the 2007 edition of the WHO classification of central nervous system tumors. All of the patients in the study received similar treatments. The inclusion criteria were as follows: i) the patients were treated with conventional therapies consisting of maximal surgical resection, followed by radiotherapy and/or chemotherapy; ii) patients received follow-up and were >18 years old. Patients who received radiotherapy or chemotherapy before admission or died from non-glioma-related diseases were excluded from this study. Areas with actively growing tumors were analyzed preferentially. Only samples that contained >80% tumor cells were selected. In cases of discrepancies, the 2 observers reviewed the slides together to achieve a consensus. Written informed consent to use both glioma and normal brain tissue and any accompanying images was obtained from the patient prior to the publication of this report. The study was performed with the approval of the Ethics Committee of Capital Medical University and was in compliance with the Helsinki Declaration.

A total of 369 glioma expression files from the cancer genome atlas (TCGA) database [9] (http://cancergenome.nih.gov), the Repository for Molecular Brain Neoplasia Data (REMBRANDT, http://caintegratorinfo.nci.nih.gov/rembrandt), and GSE16011 data [10] (http://www.ncbi.nlm.nih.gov/geo/query/acc.cgi?acc=GSE16011) were used as validation sets.

### Immunohistochemistry

Paraffin-embedded specimens were cut into 4-µm sections and baked at 65°C for 30 min. The sections were deparaffinized with xylene and rehydrated. The sections were submerged into EDTA (pH = 8.0) and autoclaved for antigen retrieval and were then treated with 3% hydrogen peroxide, followed by incubation with 1% FBS. Anti-Rab27a antibody (Abcam, ab55667) was added, and the slides were incubated overnight at 4°C. For negative controls, the primary antibody was replaced by normal mouse serum. Horseradish peroxidase (HRP)-labeled secondary antibody from the MaxVision™ HRP-Polymer antimouse immunohistochemistry (IHC) kit was applied, and the slides were incubated for 30 min at room temperature, followed by a 5-min incubation at room temperature with DAB provided in the kit for color development. Finally, the sections were counterstained with hematoxylin and mounted with Permount (BIOS, Beijing, China). The results were visualized and photographed under a light microscope. The proportion of positively stained tumor cells was graded as follows [2], [11]: 0, no positive tumor cells; 1, <5% positive tumor cells; 2, 5–20% positive tumor cells; and 3, >20% positive tumor cells. Staining intensity was recorded on a scale of 0 (no staining), 1 (weak staining, light yellow), 2 (moderate staining, yellowish brown), and 3 (strong staining, brown). The staining index was calculated as follows: staining index = staining intensity × tumor cell staining grade. High Rab27a expression was defined as a staining index score ≥4, while low expression was defined as a staining index <4. This study was approved by the Research Ethics Committee of Beijing Tiantan Hospital. Written informed consent was obtained from all patients.

### GO Analysis of Rab27a Associated Genes

After Pearson correlation analysis, GO results for the positively correlated genes (r>0.4, p<0.01) were analyzed using DAVID (http://david.abcc.ncifcrf.gov/ho me.jsp). Genes that had relative differences greater than the threshold were considered to be potentially significant, and permutations were used for the repeated measurements to estimate the false discovery rate (FDR).

### GSVA with Rab27a Expression

GSVA with Rab27a expression was analyzed using the GSVA package [12] of R [13]. The gene list was obtained online (http://amigo.geneontology.org/) (GO:0030335, positive regulation of cell migration and GO:0030336, negative regulation of cell migration).

### Statistical Analysis

For the molecular subtype annotation of the 4 datasets, we applied prediction analysis of microarrays (PAM), as previously reported [1]. Quantitative results are shown as means ± standard deviations. The difference in Rab27a expression was tested using the Student t-test for microarray data and the chi-square test for IHC results. The overall survival time (OS) was calculated from the date of diagnosis until death or the last follow-up. The survival curve of patients with high or low Rab27a expression levels was calculated with the Kaplan-Meier method, and the difference was analyzed using the two-sided log-rank test. A p-value <0.05 was considered statistically significant. All of the data analysis was performed in GraphPad Prism and R.

## Results

### The Clinical Information of Patients in CGGA

The 220 glioma patients consisted of 97 WHO Grade II gliomas (astrocytomas, oligodendrogliomas, and oligoastrocytomas), 34 WHO Grade III gliomas (anaplastic astrocytomas, anaplastic oligodendrogliomas, and anaplastic oligoastrocytomas), and 89 WHO Grade IV gliomas (GBMs). Clinical information (age, gender, preoperational Karnofsky Performance Scale [KPS] score, and treatment) was obtained from the patients’ medical records in the CGGA database (Table 1).

**Table 1:** Clinical characteristics of 220 glioma patients.

	Grade II	Grade III	Grade IV
Median age	38	39	46
Male (%)	55.7	52.9	58.4
Median KPS	90	80	80
Median OS (days)	ND	633	420
ND, not determined			

Karnofsky Performance Scale (KPS).

### The Expression of Rab27a Increased along with Grade Progression in Gliomas

**Figure 1:**
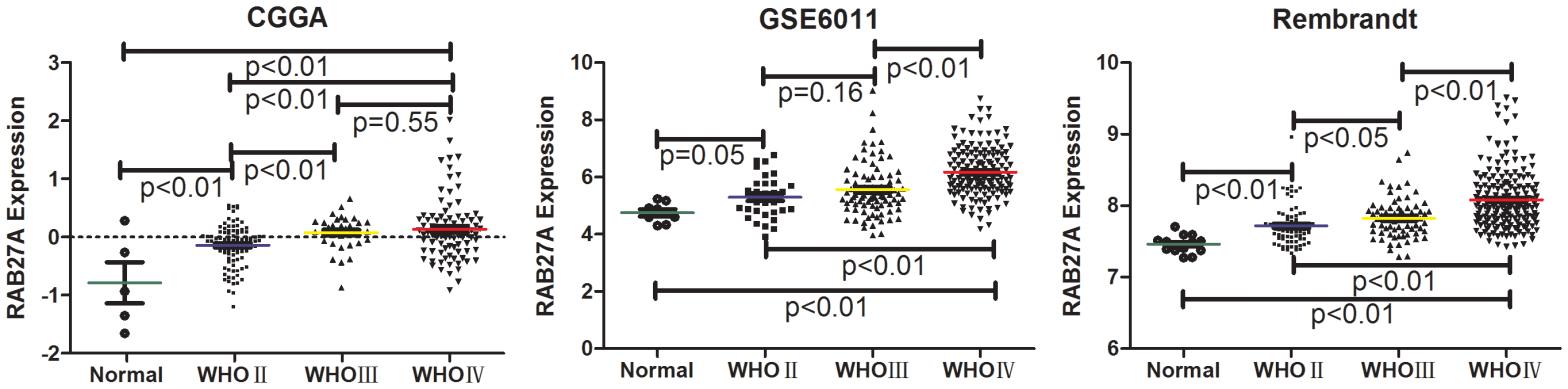
The difference in Rab27a expression in normal brain and glioma tissues in CGGA and 2 other validation datasets. We compared Rab27a expression in normal, WHO Grade II, WHO Grade III, and WHO Grade IV gliomas using the CGGA dataset (A), GSE16011 (B), and Rembrandt (C). The P values were analyzed to assess differences between the groups. A single spot represents the Rab27a expression value of an individual patient, and the lines in the middle represent the mean expression values.

By screening the differently expressed genes in the discovery dataset, we found that Rab27a expression was significantly differently between normal brain and all grades of gliomas. Rab27a expression was elevated and increased with grade progression of glioma in both the CGGA and the other 2 validation datasets (Figure 1).

### Rab27a may be a Marker of Poor Prognosis in Glioma Patients

**Figure 2:**
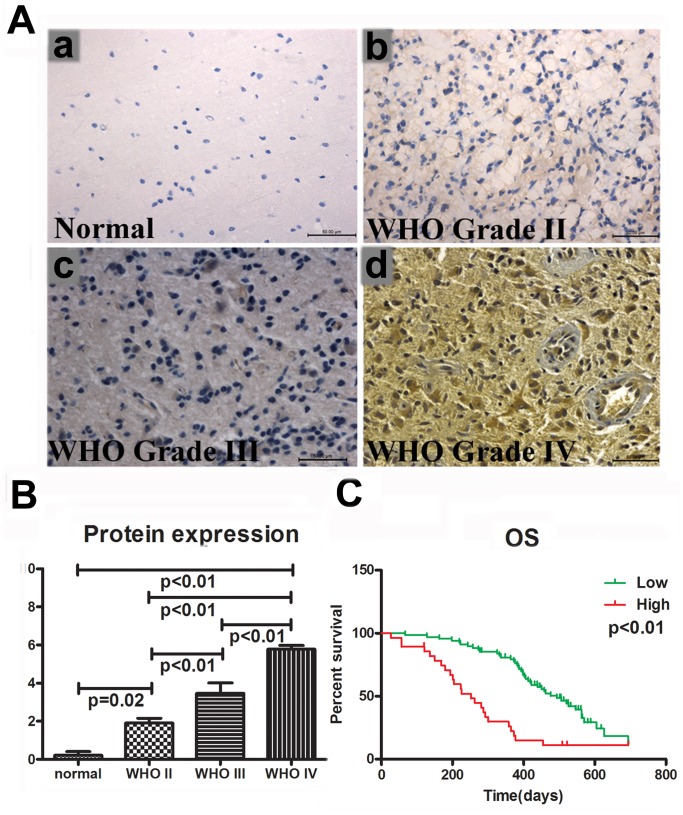
The prognostic value of Rab27a in the CGGA and validation datasets. Except for WHO Grade IV patients in the GSE16011 dataset (**I,** p = 0.28), patients from every grade could be divided into two groups with significantly different prognoses according to Rab27a expression level: **A** (p = 0.167), **B** (p = 0.160), **C** (p = 0.045) – anaplastic gliomas and GBMs in CGGA data; **D** (p<0.01)**,** **E** (p<0.01), **F** (p<0.01) – astrocytoma, anaplastic gliomas, and GBMs in Rembrandt data; **G** (p = 0.02), GBMs in TCGA data; and **H** (p<0.01), anaplastic gliomas in GSE16011 data). (**High**, higher Rab27a expression than the median. **Low**, lower Rab27a expression than the median).

We confirmed the prognosis of the 220 patients, of which 216 were used for further prognosis analysis. The prognoses of 2 patients were not available, and the OS of another 2 patients was too short, which might due to complications other than glioma. As such, these 4 patients were excluded. Both WHO Grade III glioma (Figure 2B) and WHO Grade IV glioma (Figure 2C) patients with high or low expression of Rab27a had considerably different prognoses in CGGA. The effect of Rab27a on prognosis was only marginally significant in WHO Grade II gliomas in CGGA (Figure 2A) but was significant for the WHO Grade II gliomas in the Rembrandt cohort (Figure 2D). These results were similar in the other validation datasets (Figure 2F–I).

Therefore, Rab27a was a prognostic marker in every grade of glioma. In this study, high or low expression was defined as higher or lower than the median individual in each grade.

### The Expression Status of Rab27a was Assessed in an Independent Cohort of Patients by IHC

**Figure 3:**
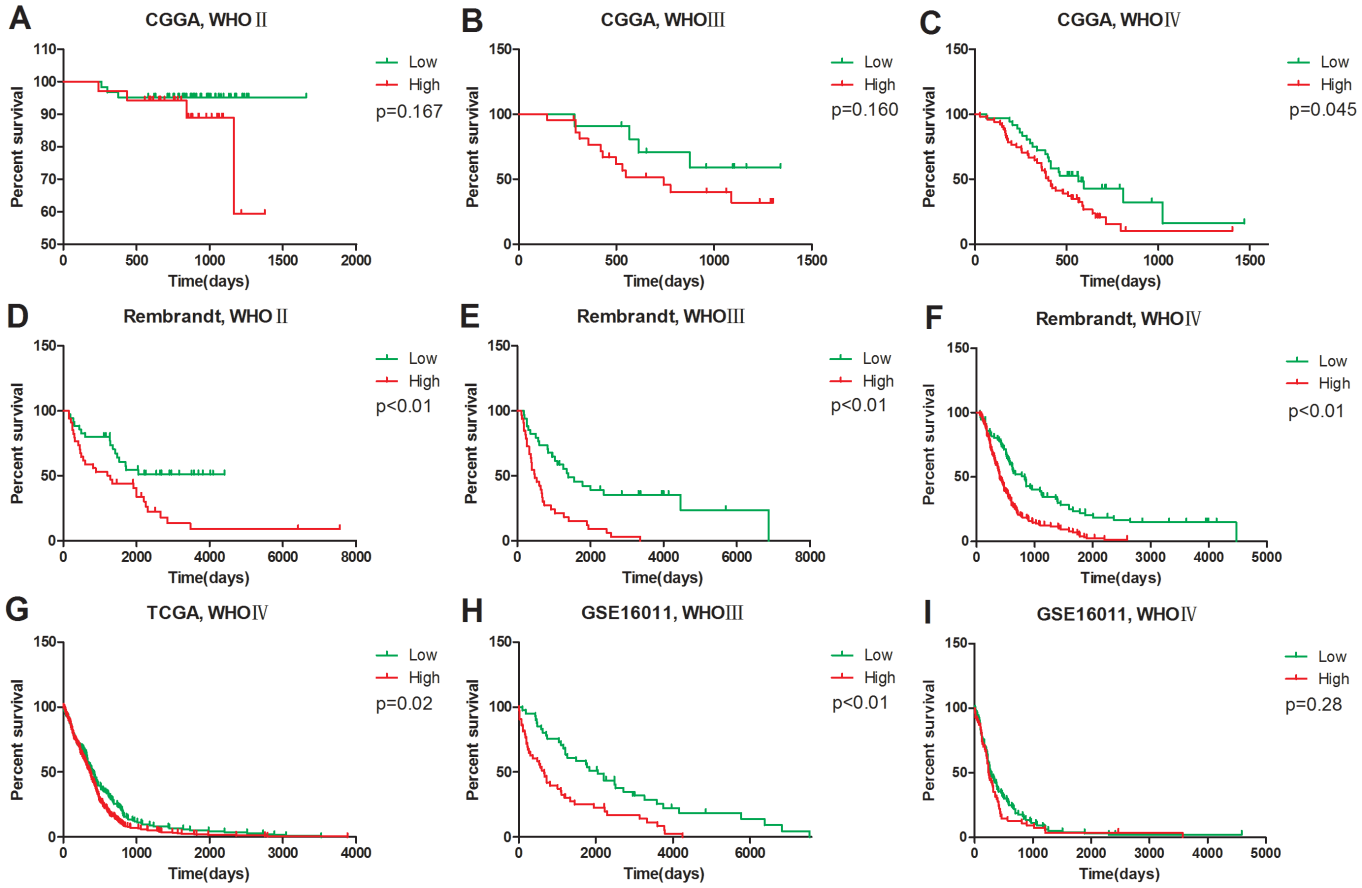
Rab27a protein expression was correlated with glioma grade progression and prognosis. Immunohistochemical staining showed that Rab27a expression increased with grade in gliomas. The sections were counterstained with hematoxylin and mounted with Permount. The immunochemistry scoring system was as follows (A): normal brain tissue: 0 (a), astrocytoma: 1 (WHO Grade II) (b), anaplastic astrocytoma: 4 (WHO Grade III) (c), and glioblastoma: 9 (WHO Grade IV) (d). (B) Histogram showed staining score increased from normal to WHO Grade IV. (C) By kaplan meier analysis including 96 GBMs, we found high Rab27b protein expression harbored significant short overall survival.

We assessed the expression status of Rab27a in an independent group of 162 glioma patients by IHC (5 normal tissues, 37 WHO Grade II gliomas, 24 WHO Grade III gliomas, and 96 GBMs) (Figure 3A). Similar to the findings above, Rab27a expression was higher in gliomas than in normal brain tissues, and the protein expression level of Rab27a increased with grade progression in gliomas, which was consistent with our findings above (Figure 3B). In the 96 CBM samples, the patients with high Rab27a expression had significantly shorter OS compared with patients with low Rab27a expression (Figure 3C). We conducted univariate Cox regression analysis using clinical and genetic variables for the independent cohorts and observed that Rab27a expression, preoperative KPS score, tumor resection extent, and IDH1 mutation status were significantly associated with OS. However, sex, age, and O6-methylguanine DNA methyltransferase (MGMT) promoter methylation status were not associated with OS (Table 2). A multivariate Cox regression analysis indicated that Rab27a was an independent prognostic factor in the independent cohort (OS: HR, 3.955; 95% CI, 2.195–7.245; p<0.001).

**Table 2:** Cox Hazard Regression Analyses of Clinicopathologic Factors and the RAB27a for Overall Survival in the Independent Cohort (GBMs, n = 96).

	Univariate analysis			Multivariate analysis		
Variable	HR	95% CI	*P*	HR	95% CI	*p*
Sex	0.853	0.526–1.382	0.518			
Age	0.940	0.580–1.522	0.800			
PreoperativeKPS score	0.556	0.343–0.901	0.017	0.728	0.434–1.220	0.228
Extent ofresection	0.636	0.379–1.067	0.086	0.809	0.467–1.404	0.451
IDH1mutation	0.302	0.155–0.585	<0.001	0.237	0.117–0.480	<0.001
MGMTpromotermethylation	0.908	0.442–1.866	0.933			
RAB27a	3.321	1.983–5.561	<0.001	3.955	2.159–7.245	<0.001

### The Expression of Rab27a showed a Subtype Preference

**Figure 4:**
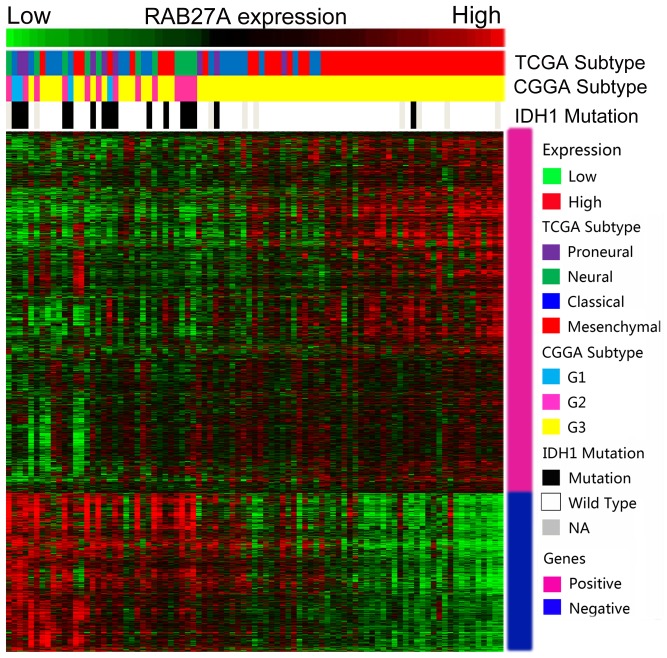
Rab27a expression showed mesenchymal, G3 subtype, and IDH1 wild-type preferences. For each patient, the TCGA and CGGA subtypes are annotated as previously reported and are listed in the upper part together with the IDH1 mutation status, which was obtained from the CGGA database. Correlated genes are supervised by clustering according to Rab27a expression, from low to high. The positively and negatively correlated genes are shown in the lower panel (marked pink and blue on the right, respectively). NA, not available.

**Figure 5:**
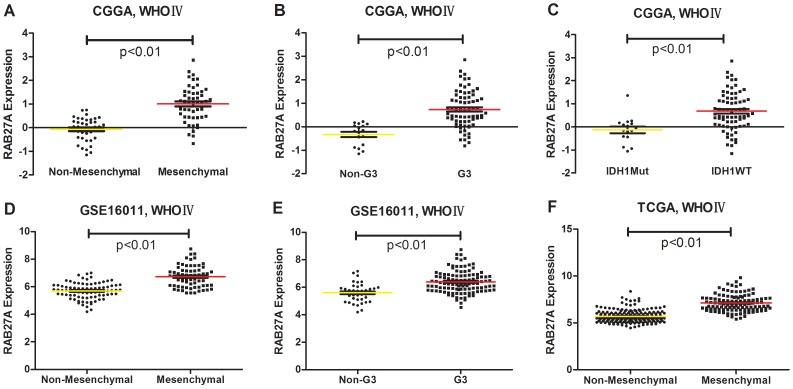
Rab27a expression was significantly different for different TCGA and CGGA subtypes and IDH1 mutation status. A (p<0.01), D (p<0.01), and F (p<0.01) indicate that Rab27a has a mesenchymal preference. B (p<0.01) and E (p<0.01) indicate that Rab27a has a G3 preference. C (p<0.01) indicates that patients with wild type IDH1 also had higher Rab27a expression compared to patients with mutations in the IDH1 gene. A single spot represents the Rab27a expression value of an individual patient, and the lines in the middle represent the mean expression value. The difference in Rab27a expression was tested by the Student t-test.

As Rab27a expression was associated with high-grade gliomas and worse prognosis, we screened its expression in different molecular subtypes of WHO Grade IV gliomas. As previously reported, we annotated the 4 datasets from TCGA and CGGA classification systems using PAM [1], [9]. Rab27a exhibited mesenchymal and G3 subtype preferences. Patients with wild-type IDH1 had higher Rab27a expression than patients with mutations in the IDH1 gene (Figure 4). The expression difference is shown in Figure 5.

### Rab27a was Mainly Associated with Cell Migration

Pearson correlation analysis was used to identify significantly positively correlated genes (2932 genes marked in pink in Figure 4) from CGGA, which were subsequently used for GO analysis. The 1285 genes marked in blue were negatively correlated with Rab27a expression. GO analysis revealed that, among the genes positively correlated to Rab27a, genes related to cell migration were significantly enriched in cases with high Rab27a expression (Table 3). Using a p value of <0.001, more than 30 biologic processes mainly related to cell migration were significantly enriched in the WHO Grade IV samples with high Rab27a expression.

**Table 3:** Gene sets enriched in WHO Grade IV glioma samples with high Rab27A expression.

NAME	COUNT	FOLD ENRICHMENT	P Value	FDR
GO:0009611∼response to wounding	177	6.68E-35	1.25E-31	2.566955
GO:0007155∼cell adhesion	171	2.95E-17	5.52E-14	1.877669
GO:0022610∼biological adhesion	171	3.41E-17	6.38E-14	1.87499
hsa04512:ECM-receptor interaction	42	2.14E-12	2.64E-09	3.089307
GO:0030198∼extracellular matrix organization	43	2.49E-12	4.66E-09	3.178016
hsa04510:Focal adhesion	70	9.05E-11	1.12E-07	2.151756
GO:0031589∼cell-substrate adhesion	39	1.15E-10	2.16E-07	3.058859
GO:0042060∼wound healing	59	2.04E-10	3.82E-07	2.374322
GO:0007229∼integrin-mediated signaling pathway	31	5.96E-10	1.12E-06	3.403961
GO:0016477∼cell migration	74	1.13E-09	2.11E-06	2.060837
GO:0007160∼cell-matrix adhesion	35	1.73E-09	3.24E-06	3.022727
GO:0032103∼positive regulation of response to external stimulus	27	3.18E-08	5.96E-05	3.242685
GO:0043062∼extracellular structure organization	48	6.27E-08	1.17E-04	2.263469
hsa04514:Cell adhesion molecules (CAMs)	47	8.36E-08	1.03E-04	2.199961
GO:0051272∼positive regulation of cell motion	34	1.09E-07	2.03E-04	2.666698
GO:0030155∼regulation of cell adhesion	42	1.41E-07	2.64E-04	2.356403
GO:0030335∼positive regulation of cell migration	31	3.97E-07	7.43E-04	2.677273
GO:0030334∼regulation of cell migration	47	5.48E-07	0.001025	2.137628

### Correlation between Migration Genes and Rab27a

**Figure 6:**
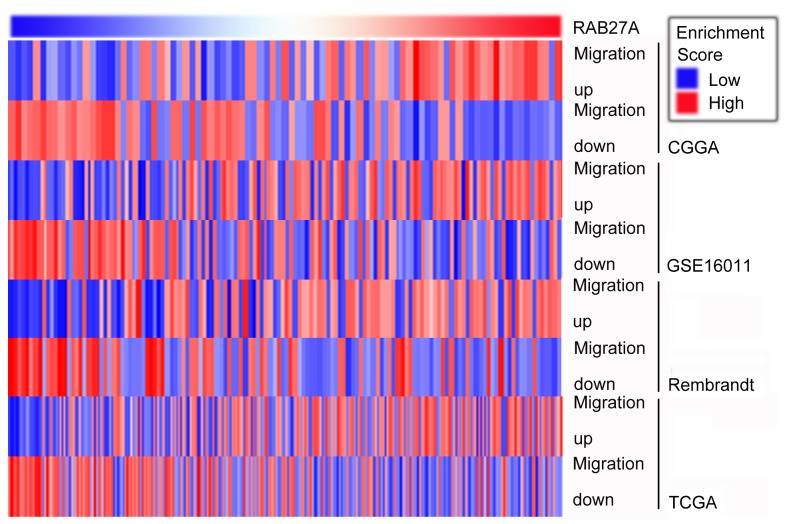
Gene set variation analysis with Rab27a expression. Gene set variation analysis with Rab27a expression was analyzed using the GSVA package. Gene expression signatures of migration were generated from the GO list. Rab27a expression is listed from left to right, starting with the highest expression level. A high enrichment score indicates a positive correlation with Rab27a expression, and a low enrichment score indicates the reverse.

As GO analysis showed that Rab27a expression was strongly associated with migration, we performed GSVA with Rab27a expression (Figure 6). The genes up- and down-regulated in migration correlated positively and negatively, respectively, with Rab27a expression in CGGA and the other 3 validation datasets.

## Discussion

Glioma is the most common type of brain tumor and is an important cause of cancer-related mortality among adults and children [1]. The invasiveness of glioma cells is a major cause of therapeutic failure, especially in GBM [14], [15], [16]. With the development of high-throughput technologies, we are able to easily explore gene expression and function.

Rab GTPases are master regulators of intracellular trafficking and in recent years, their roles in the control of different aspects of tumor progression have emerged. Central roles of Rab GTPases that have been reported include cell migration, invasion, proliferation, communication with stromal cells, and the development of drug resistance. Some Rabs, such as Rab1b, Rab4b, Rab10, Rab22a, Rab24, and Rab25, are dysregulated in many cancers [17], [18], [19], [20]. However, there have only been a few reports about Rab GTPases in glioma [8], [21].

Rab27a is the only member of the Rab family whose mutation has been shown to result in a hereditary disease, specifically Griscelli syndrome. Rab27a expression is frequently altered in many tumor types [4], [5], [22], [23]. Both in vivo and in vitro studies have demonstrated the role of Rab27a in promoting proliferation and invasion and suppressing apoptosis, processes that are associated with cancer metastasis and progression.

Only two reports have studied the role of Rab27a in promoting proliferation and invasion and suppressing apoptosis in gliomas [7], [8]. However, these two studies only used functional assays in GBM cell lines, and the comprehensive effect of Rab27a on the occurrence and development of glioma and its expression patterns in glioma tissues was still unclear. In the present study, we found that Rab27a was highly expressed in gliomas compared to normal brain tissues in an mRNA microarray dataset from the CGGA (Figure 1). In glioma tissues, the mRNA expression level increased with grade progression. The expression difference was validated by microarray data from other datasets and IHC results from an independent group of patients from the CGTD. These results indicated that Rab27a is a potential marker for grading gliomas. Genes that were positively correlated with Rab27a in CGGA were analyzed by GO, and the results indicated Rab27a is involved in promoting proliferation, migration, cell activation, and angiogenesis and in the negative regulation of apoptosis. Promotion of migration was the most significant category obtained from the GO results (Table 3), which was confirmed by GSVA of 4 datasets. Rab27a has been suggested as a possible therapeutic target in cancer cells. However, the other characteristics of Rab27a, especially its contribution to grade progression and tumor survival rate, make such strategies less appealing. Improved knowledge of the downstream mediators of the effects of Rab27a in cancer cells may allow for the dissection of the different Rab27a-induced phenotypes and, thus, the generation of specific targeted therapies.

The Kaplan-Meier method was used for prognosis analysis, and we generated survival curves using data from CGGA and 3 other datasets. We found that patients with higher Rab27a expression had significantly worse prognoses. This observation was true for patients with all grades of glioma, which demonstrated the predominance of the detrimental effects of Rab27a over its beneficial effects in gliomas. Hence, Rab27a is likely to be a better prognostic marker than a therapeutic target. We also found an association between Rab27a expression and mesenchymal subtype, G3 subtype, and IDH1 mutation patients, which was in concordance with the poor prognosis of patients with high Rab27a expression.

In summary, Rab27a expression was significantly associated with grade progression and poor prognosis in all glioma grades, including mesenchymal and G3 subtype and wild-type IDH1, suggesting Rab27a as a novel biomarker with potentially important therapeutic implications.
